# Sex differences in the relationship between serum total bilirubin and risk of incident metabolic syndrome in community-dwelling adults: Propensity score analysis using longitudinal cohort data over 16 years

**DOI:** 10.1186/s12933-024-02182-6

**Published:** 2024-03-11

**Authors:** Ae Hee Kim, Da-Hye Son, Mid-Eum Moon, Soyoung Jeon, Hye Sun Lee, Yong-Jae Lee

**Affiliations:** 1 Department of Family Medicine, Gangnam Severance Hospital, Yonsei University College of Medicine, Seoul, Korea; 2 Biostatistics Collaboration Unit, Yonsei University College of Medicine, Seoul, Korea; 3 Department of Family Medicine, Yonsei University College of Medicine Gangnam Severance Hospital, 211 Eonju-ro, Gangnam-gu, Seoul, 06273 Korea

## Abstract

**Background:**

Research on identifiable risks for metabolic syndrome (MetS) is ongoing, and growing evidence suggests that bilirubin is a potent antioxidant and cytoprotective agent against MetS. However, there have been conflicting results on the association between bilirubin and MetS. Our study aimed to validate the association by separately stratifying data for men and women in a longitudinal prospective study.

**Methods:**

Data were derived from the Korean Genome Epidemiology Study provided by the Korea Centers for Disease Control and Prevention. Data from 5,185 adults aged 40–69 years (3,089 men and 2,096 women) without MetS were analyzed. The participants were divided according to sex-specific quartiles of serum total bilirubin levels and followed up biennially for 16 years (until 2018). The log-rank test was used for obtaining the Kaplan-Meier curves of cumulative incidence of MetS according to sex-specific serum total bilirubin quartiles, and the hazard ratios (HRs) with 95% confidence intervals (CIs) for incident metabolic syndrome were analyzed with a multiple Cox proportional hazard regression analysis model, after propensity score matching for removing differences at baseline.

**Results:**

With increasing serum total bilirubin quartiles, the incidence rate per 1000 person-years proportionally decreased in both men and women. After propensity score matching and adjusting for confounding variables, the HRs (95% CIs) for MetS of the highest quartile in reference to the lowest quartile were 1.00 (0.80–1.24) for men and 0.80 (0.65–0.99) for women. Higher quartiles of serum total bilirubin showed significantly lower cumulative incidence of MetS in women (log-rank test *p* = 0.009), but not in men (log-rank test *p* = 0.285).

**Conclusion:**

Serum total bilirubin levels were significantly inversely associated with MetS in women, but there was no significant association observed in men. Sex differences in the effects of serum total bilirubin should be noted when predicting incident MetS by sex in clinical settings.

## Introduction

Metabolic syndrome (MetS) is a cluster of interrelated risk factors for cardiovascular disease (CVD) and type 2 diabetes (T2DM), such as elevated blood pressure, dyslipidemia, elevated fasting glucose, and central obesity [1]. There is a general consensus that the global prevalence of MetS has been continuously increasing worldwide in recent decades [2]. For example, in the United States, the prevalence of MetS increased from 37.6% in 2011–2012 to 41.8% in 2017–2018 [3]. Similarly, in Korea, the prevalence of age-adjusted MetS increased from 24.5% in 2008 to 28.1% in 2017 and continues to rise [4]. These upward trends in MetS are associated with increased risks of atherosclerotic CVD, T2DM, and all-cause mortality, and the trends are expected to increase even further in the future [5]. In addition, the detrimental effects on public health burden and medical care financial costs are also becoming inevitable issues to be resolved. Thus, early identification of risk factors for MetS is crucial.

Growing evidence suggests that bilirubin is a potent antioxidant and a cytoprotective agent against cardiometabolic diseases [6–8]. Many studies have already been published regarding the roles of bilirubin on insulin resistance and MetS [9–13]. Generally, cross-sectional studies have reported an inverse association between bilirubin level and MetS. Some prospective studies have reported similar findings, but others have shown different results. These conflicting results suggest that inverse association findings are inconclusive. According to a recent meta-analysis by Liang et al. [9]. , the association between bilirubin level and MetS was significant in the combined pooled odds ratio (OR) of seven cross-sectional studies (OR = 0.91, 95% CI = 0.70–0.94, *P* = 0.005). However, the combined pooled OR of five cohort studies was not significant when confined to only men (OR = 0.91, 95% CI = 0.54–1.53, *P* = 0.72). In women only, the combined pooled OR of seven cross-sectional studies was significant (OR = 0.69, 95% CI = 0.57–0.84, *P* = 0.0002), but the combined pooled OR of two cohort studies was not significant (OR = 1.28, 95% CI = 0.60–1.02, *P* = 0.68). In five studies that were not stratified according to sex, the combined pooled OR was significant for an inverse association between bilirubin and MetS (OR = 0.75, 95% CI = 0.61–0.91, *P* = 0.004). Due to these conflicting results, our longitudinal prospective study with 16 years of follow-up aimed to analyze the effects of bilirubin on risk of incident MetS by separately stratifying for men and women.

## Methods

### Study population

The data in this study were derived from the Korean Genome and Epidemiology Study (KoGES) provided by the Korea Centers for Disease Control and Prevention (http://www.cdc.go.kr/CDC/eng/main.jsp). The database consists of the results from six large prospective cohort studies governed by the Korea National Institute of Health for investigating factors associated with chronic diseases in Korea. We used the Ansan-Ansung cohort study which involves community dwellers of both sexes aged 40 to 69 years who either live in Ansan (an urban area) or Ansung (a rural area). These participants were enrolled in 2001–2002 and have been assessed biennially until 2018. Informed consent was obtained from all participants, and participation in the study was voluntary. Our study was conducted in accordance with the Declaration of Helsinki and was approved by the Ethics Committee of the Korean Health and Genomic Study at the Korea National Institute of Health. Detailed information on KoGES has been published in previous reports [14]. The Ansan-Ansung study protocol was reviewed and approved by the Institutional Review Board of the Korea Centers for Disease Control and Prevention, and all study participants provided written informed consent. This study was approved by the Institutional Review Board of Gangnam Severance Hospital (IRB number: 3-2018-0348).

**Fig. 1:**
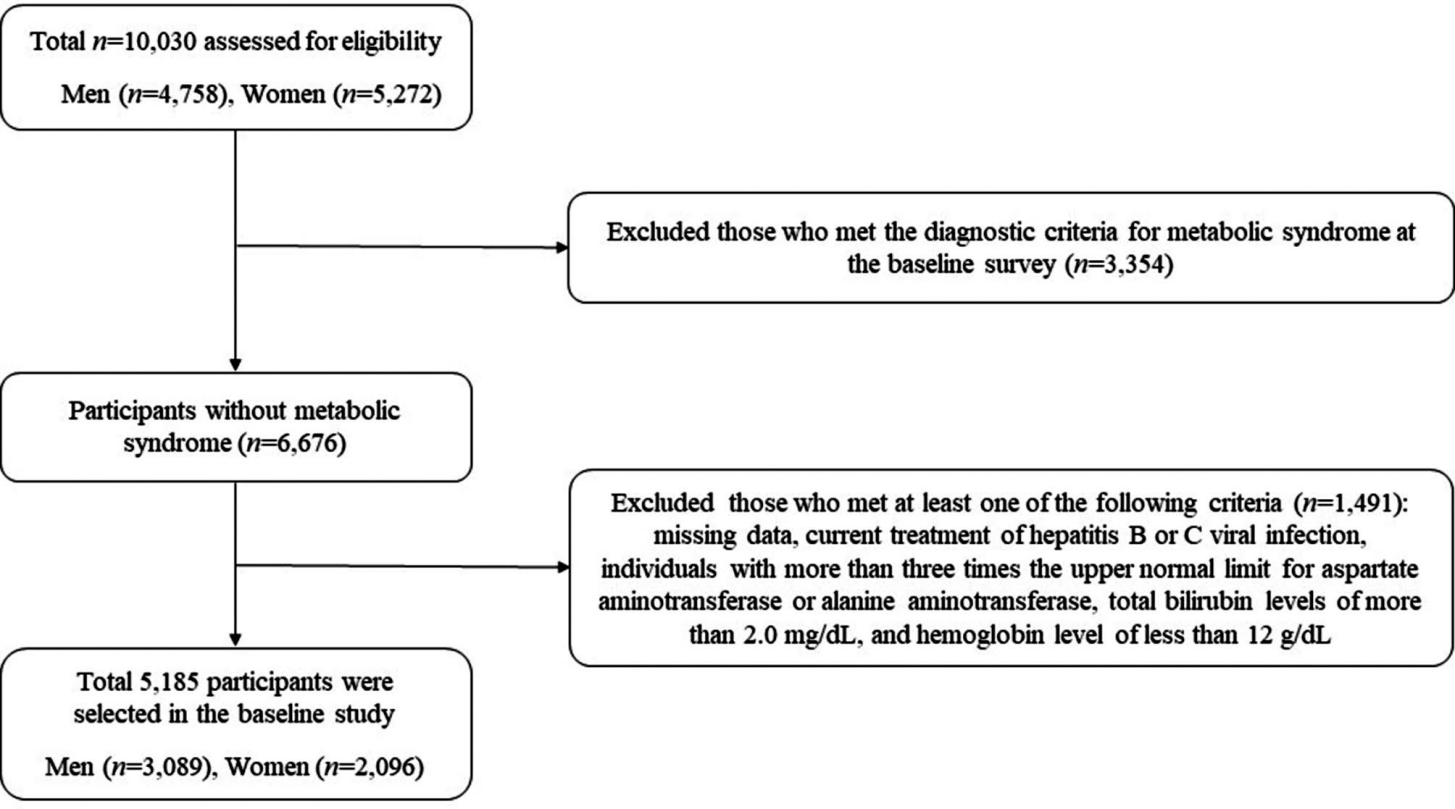
Flow chart for selection of the study population

At baseline, 10,030 participants (4,758 men and 5,272 women) were assessed for eligibility (Fig. 1). We excluded those who already met the criteria for MetS at baseline (*n* = 3,354). In addition, participants who met at least one of the following criteria were excluded (*n* = 1,491): (1) having missing data, (2) being currently treated for hepatitis B and/or hepatitis C viral infection, (3) having more than three times the upper normal limit for aspartate aminotransferase (AST) and/or alanine aminotransferase (ALT), (4) having a total bilirubin level of more than 2.0 mg/dL, and (5) having a hemoglobin level of less than 12 g/dL. After employing these criteria, 5,185 participants (3,089 men and 2,096 women) were selected for participation in our study.

### Definition of metabolic syndrome

We defined MetS according to the modified National Cholesterol Education Program Adult Treatment Panel III [15], except for its waist circumference provisions. For waist circumference, we had to adjust for ethnic differences [16]. MetS included having any three of the following five conditions: (1) waist circumference (WC) of ≥ 90 cm in men and ≥ 80 cm in women, (2) triglyceride level of ≥ 150 mg/dL or under current triglyceride-lowering drug treatment, (3) high-density lipoprotein cholesterol (HDL-C) level of < 40 mg/dL in men and < 50 mg/dL in women, (4) systolic blood pressure (BP) of ≥ 130 mmHg and/or diastolic BP of ≥ 85 mmHg or on drug treatment for hypertension, and (5) fasting glucose level of ≥ 100 mg/dL or on current glucose-lowering drug treatment.

### Measurement of anthropometric and biochemical parameters

Anthropometric measurements were obtained by trained medical staff following standardized procedures. Body weight was measured with a digital electronic scale that was set to 0 prior to obtaining measurements to the nearest 0.1 kg while participants wore light indoor clothing without shoes. Height was measured with a rod attached to a balanced beam scale (Seca 225) using a Frankfurt horizontal plane to the nearest 0.1 cm while participants stood as straight as possible and maintained deep breath inspiration. WC was measured to the nearest 0.1 cm by a trained technician in a horizontal plane at a level midway between the lower rib margin and the iliac crest following a normal expiration. Body mass index (BMI, kg/${\text{m}}^{2}$) was defined as the ratio of weight (kg) divided by height squared (${\text{m}}^{2}$). A current smoker was defined as a participant who smoked 100 cigarettes in his or her lifetime and continues to smoke. Regular drinker was defined as a participant who consumed alcohol more than once a month. Regular exercise was defined as engaging in moderate intensity physical exercise more than three times per week. Systolic and diastolic BP measurements were conducted using a standard mercury sphygmomanometer (Baumanometer, Baum Co. Inc., N.Y.), and the mean of the two arm readings was used for the analysis. The mean arterial BP was calculated as: [systolic BP + (2 x diastolic BP)]/3. The fasting plasma glucose, postprandial plasma glucose, glycosylated hemoglobin A1c (HbA1c), total cholesterol, triglyceride, high density lipoprotein cholesterol (HDL-C), aspartate aminotransferase (AST), alanine aminotransferase (ALT), and total bilirubin levels were all measured enzymatically with a 747 Chemistry Analyzer (Hitachi 7600, Tokyo, Japan). Overnight fasting for at least eight hours was conducted prior to performing these tests. Homeostatic model assessment for insulin resistance (HOMA-IR) was calculated as: [fasting insulin ($\mu$IU/mL) x fasting glucose (mg/dL)/405] [17].

### Statistical analysis

The data in our study are presented as mean and standard deviation, median with interquartile range, or number with percentage. Our participants were divided according to quartiles of serum total bilirubin levels in each sex as: Q1: ≤ 0.46, Q2: 0.47–0.62, Q3: 0.63–0.82, and Q4: ≥ 0.83 mg/dl for men; and Q1: ≤ 0.38, Q2: 0.39–0.49, Q3: 0.50–0.65, and Q4: ≥ 0.66 mg/dl for women. The baseline characteristics of the study population were compared using chi-squared tests for categorical variables with normal distributions and analysis of variance for continuous variables with normal distributions, or the Kruskal-Wallis test for continuous variables without normal distributions. Distribution normality was evaluated for skewness using the Kolmogorov-Smirnov test. HOMA-IR, triglyceride, AST, and ALT were expressed as median with interquartile range in descriptive analysis due to skewed distributions. The remaining continuous data were expressed as mean and standard deviation. To reduce impact of selection bias and potential confounding effects between different bilirubin groups at baseline, we performed propensity score matching (matched for age, fasting plasma glucose, systolic BP, diastolic BP, WC, triglyceride, and HDL-C). For obtaining the Kaplan-Meier curves of cumulative incidence of MetS according to sex-specific serum total bilirubin quartiles, the log-rank test was used. Multivariate Cox proportional hazard regression models were used to calculate the hazard ratios (HRs) and 95% CIs for incident MetS after setting the lowest quartile as the reference group and adjusting for potential confounding variables. All analyses were conducted using SAS statistical software (version 9.4; SAS Institute Inc., Cary, NC, USA), and all P-values were two-tailed with statistical significance set at *P* < 0.05.

## Results

Baseline characteristics of the 5,185 study participants and p-values according to each quartile of serum total bilirubin level before and after propensity score matching in men are described in Table 1 and women in Table 2. Before matching, as serum total bilirubin quartile increased, postprandial plasma glucose also increased in both sexes; however, fasting plasma glucose, total cholesterol, HDL-C, regular drinker percentage, and family history of diabetes increased only in men. With increasing serum total bilirubin quartiles, age, HbA1c, and current smoker percentage decreased in both sexes; regular exercise decreased only in men, and WC, systolic BP, diastolic BP, mean BP, HOMA-IR, and triglyceride level decreased only in women. After matching, MetS related factors that were significantly different between bilirubin quartile groups became nonsignificant.

**Table 1:** Baseline characteristics of study population by serum total bilirubin quartiles in men before and after propensity score matching

	Total bilirubin quartiles in men (Before matching)					Total bilirubin quartiles in men (After matching)				
	Q1 (≤ 0.46)	Q2 (0.47–0.62)	Q3 (0.62–0.82)	Q4 (≥ 0.83)	P-value	Q1 (≤ 0.46)	Q2 (0.47–0.62)	Q3 (0.62–0.82)	Q4 (≥ 0.83)	P-value
n	796	781	754	758		422	422	422	422	
Age (years)	54.0 (9.0)	51.7 (8.9)	50.8 (8.4)	49.6 (8.5)	< 0.001	49.5 (7.6)	49.8 (8.2)	49.0 (7.2)	49.6 (8.1)	0.517
Waist circumference (cm)	81.2 (6.8)	81.5 (6.8)	81.7 (6.4)	81.6 (6.8)	0.550	82.2 (6.6)	81.7 (6.7)	81.7 (6.3)	81.1 (6.9)	0.144
Systolic BP (mmHg)	119.5 (16.0)	118.8 (16.0)	117.8 (15.4)	118.7 (16.6)	0.169	117.5 (14.5)	118.4 (15.8)	116.3 (14.7)	117.9 (16.6)	0.232
Diastolic BP (mmHg)	79.6 (10.4)	79.6 (10.4)	79.3 (10.1)	80.4 (10.9)	0.244	79.7 (10.0)	79.9 (11.1)	79.0 (10.1)	79.7 (10.8)	0.625
Mean arterial BP (mmHg)	92.9 (11.2)	92.7 (11.6)	92.1 (11.4)	93.2 (12.2)	0.368	92.3 (10.9)	92.7 (12.1)	91.4 (11.1)	92.4 (12.1)	0.403
Fasting plasma glucose (mg/dL)	82.7 (10.5)	87.1 (17.9)	87.3 (17.8)	87.6 (17.7)	< 0.001	85.0 (9.3)	85.7 (9.9)	85.8 (9.5)	84.7 (9.4)	0.298
Postprandial plasma glucose (mg/dL)	106.8 (35.8)	113.9 (47.0)	116.9 (46.4)	115.8 (41.1)	< 0.001	105.9 (33.4)	112.6 (41.3)	113.8 (40.0)	112.6 (34.6)	0.009
HbA1c	5.67 (0.58)	5.73 (0.81)	5.64 (0.67)	5.56 (0.69)	< 0.001	5.6 (0.4)	5.6 (0.5)	5.5 (0.4)	5.5 (0.4)	< 0.001
HOMA-IR	1.26 (0.95–1.77)	1.32 (0.97–1.82)	1.30 (0.99–1.78)	1.20 (0.88–1.65)	0.001	1.4 (1.1–1.9)	1.4 (1.0-1.9)	1.3 (1.0-1.7)	1.2 (0.9–1.6)	< 0.001
Total cholesterol (mg/dL)	182.3 (32.1)	192.1 (35.4)	191.2 (35.1)	193.5 (34.9)	< 0.001	187.7 (33.3)	192.1 (33.6)	192.2 (34.7)	192.2 (33.8)	0.145
Triglyceride (mg/dL)	127 (98–174)	130 (102–180)	134 (101–176)	123 (93–166)	0.002	129.5 (98–174)	128.5 (102–180)	134 (102–185)	123 (95–169)	0.108
HDL-cholesterol (mg/dL)	44.7 (9.8)	45.1 (9.6)	45.2 (9.8)	47.3 (10.1)	< 0.001	45.7 (10.0)	45.4 (10.0)	44.8 (9.4)	46.4 (9.9)	0.105
AST (U/L)	28 (24–33)	27 (24–33)	28 (24–33)	29 (24–35)	< 0.001	28 (25–34)	27.5 (24–33)	28 (24–33)	29 (24–36)	0.028
ALT (U/L)	25 (19–33)	26 (20–33)	25 (20–34)	27 (20–35)	0.145	27 (20–37)	26 (20–35)	25 (20–33)	27 (20–36)	0.186
Current smoker (%) ^a^	65.3	53.2	45.7	37.4	< 0.001	64.4	54.4	46.9	35.6	0.011
Regular drinker (%) ^b^	67.0	69.9	72.6	77.5	< 0.001	73.4	72.1	73.5	76.3	0.272
Regular exercise (%) ^c^	30.6	28.8	29.4	23.9	0.013	28.1	31.1	31.2	24.1	0.367
Family history of diabetes (%)	7.4	10.0	10.9	12.4	0.007	9.5	9.7	11.6	11.8	0.907

Data are expressed as the mean (SD), median (IQR) or percentage

Propensity score matched for age (years), fasting plasma glucose (mg/dL), systolic BP (mmHg), diastolic BP (mmHg), waist circumference (cm), triglyceride (mg/dL), HDL-cholesterol (mg/dL)

Abbreviations: BP, blood pressure; HbA1c, glycated hemoglobin; HOMA-IR, homeostatic model assessment for insulin resistance; HDL, high density lipoprotein; AST, aspartate aminotransferase; ALT, alanine aminotransferase

P-values were calculated with the use of ANOVA-test or chi-square test

^a^An adult who has smoked 100 cigarettes in his or her lifetime and who currently smokes cigarettes

^b^Alcohol intake ≥ once/month

^c^Moderate intensity physical exercise ≥ three times/week

**Table 2:** Baseline characteristics of study population by serum total bilirubin quartiles in women before and after propensity score matching

	Total bilirubin quartiles in women (Before matching)					Total bilirubin quartiles in women (After matching)				
	Q1 (≤ 0.38)	Q2 (0.39–0.49)	Q3 (0.50–0.65)	Q4 (≥ 0.66)	P-value	Q1 (≤ 0.38)	Q2 (0.39–0.49)	Q3 (0.50–0.65)	Q4 (≥ 0.66)	P-value
n	544	510	532	510		326	326	326	326	
Age (years)	52.5 (8.8)	51.5 (8.5)	49.8 (8.3)	48.9 (8.1)	< 0.001	49.2 (7.4)	49.5 (8.1)	49.3 (7.6)	49.2 (8.0)	0.957
Waist circumference (cm)	79.7 (8.7)	78.5 (8.6)	77.6 (8.8)	76.9 (8.2)	< 0.001	77.7 (7.7)	77.3 (7.9)	77.5 (8.0)	77.7 (7.9)	0.912
Systolic BP (mmHg)	116.8 (16.7)	115.3 (16.5)	114.5 (17.0)	110.9 (14.8)	< 0.001	112.7 (14.2)	114.2 (15.4)	114.5 (16.9)	112.0 (13.8)	0.1
Diastolic BP (mmHg)	76.0 (10.0)	75.9 (10.4)	75.7 (10.9)	74.0 (9.7)	0.005	74.9 (9.4)	76.0 (9.9)	76.0 (11.2)	74.6 (9.3)	0.129
Mean arterial BP (mmHg)	89.6 (11.6)	89.0 (11.9)	88.6 (12.5)	86.3 (10.9)	< 0.001	87.5 (10.5)	88.7 (11.3)	88.8 (12.7)	87.1 (10.3)	0.104
Fasting plasma glucose (mg/dL)	81.3 (8.5)	81.6 (12.6)	81.2 (11.6)	81.0 (13.2)	0.862	81.3 (9.3)	81.5 (9.8)	81.3 (13.3)	81.1 (13.2)	0.98
Postprandial plasma glucose (mg/dL)	114.5 (30.1)	117.3 (35.8)	120.3 (32.0)	120.4 (36.2)	0.010	113.3 (30.0)	116.0 (33.2)	119.8 (30.7)	121.9 (38.3)	0.005
HbA1c	5.60 (0.55)	5.58 (0.62)	5.49 (0.49)	5.45 (0.62)	< 0.001	5.6 (0.5)	5.5 (0.4)	5.5 (0.5)	5.5 (0.6)	0.027
HOMA-IR	1.48 (1.14–2.06)	1.41 (1.04–1.86)	1.35 (1.01–1.72)	1.27 (0.87–1.74)	< 0.001	1.5 (1.1–2.1)	1.4 (1.0-1.9)	1.4 (1.0-1.7)	1.2 (0.9–1.7)	< 0.001
Total cholesterol (mg/dL)	185.9 (32.0)	188.7 (33.6)	189.6 (35.1)	189.0 (34.6)	0.304	182.4 (32.5)	187.8 (34.5)	189.0 (34.0)	189.8 (36.5)	0.028
Triglyceride (mg/dL)	111 (90–140)	109 (86–135)	104 (85–131)	102 (83–128)	0.001	106.0 (87–130)	109.0 (84–134)	103.0 (88–128)	104.0 (86–131)	0.443
HDL-cholesterol (mg/dL)	48.9 (10.1)	49.3 (9.9)	49.9 (9.9)	49.0 (10.2)	0.412	48.9 (10.0)	49.5 (9.7)	49.4 (9.9)	48.3 (9.7)	0.348
AST (U/L)	24 (21–28)	25 (21–28)	24 (21–28)	24 (21–30)	0.449	23.0 (21–27)	24.0 (21–28)	24.0 (22–28)	24.0 (22–30)	< 0.001
ALT (U/L)	19 (16–24)	19 (16–24)	18 (15–24)	19 (15–24)	0.457	18.0 (15–24)	18.5 (15–22)	18.0 (16–22)	19.0 (16–24)	0.212
Current smoker (%) ^a^	5.71	3.8	2.5	1.8	< 0.001	6.3	3.1	2.8	0.9	0.02
Regular drinker (%) ^b^	27.9	26.9	34.5	31.4	0.022	32.3	28.5	33.3	30.2	0.463
Regular exercise (%) ^c^	21.5	23.2	24.2	28.0	0.071	22.3	24.2	25.7	28.6	0.3
Family history of diabetes (%)	10.3	12.9	14.5	14.5	0.094	11.3	14.4	16.0	12.9	0.242

Data are expressed as the mean (SD), median (IQR) or percentage

Propensity score matched for age (years), fasting plasma glucose (mg/dL), systolic BP (mmHg), diastolic BP (mmHg), waist circumference (cm), triglyceride (mg/dL), HDL-cholesterol (mg/dL)

Abbreviations: BP, blood pressure; HbA1c, glycated hemoglobin; HOMA-IR, homeostatic model assessment for insulin resistance; HDL, high density lipoprotein; AST, aspartate aminotransferase; ALT, alanine aminotransferase

P-values were calculated with the use of ANOVA-test or chi-square test

^a^An adult who has smoked 100 cigarettes in his or her lifetime and who currently smokes cigarettes

^b^Alcohol intake ≥ once/month

^c^Moderate intensity physical exercise ≥ three times/week

**Fig. 2:**
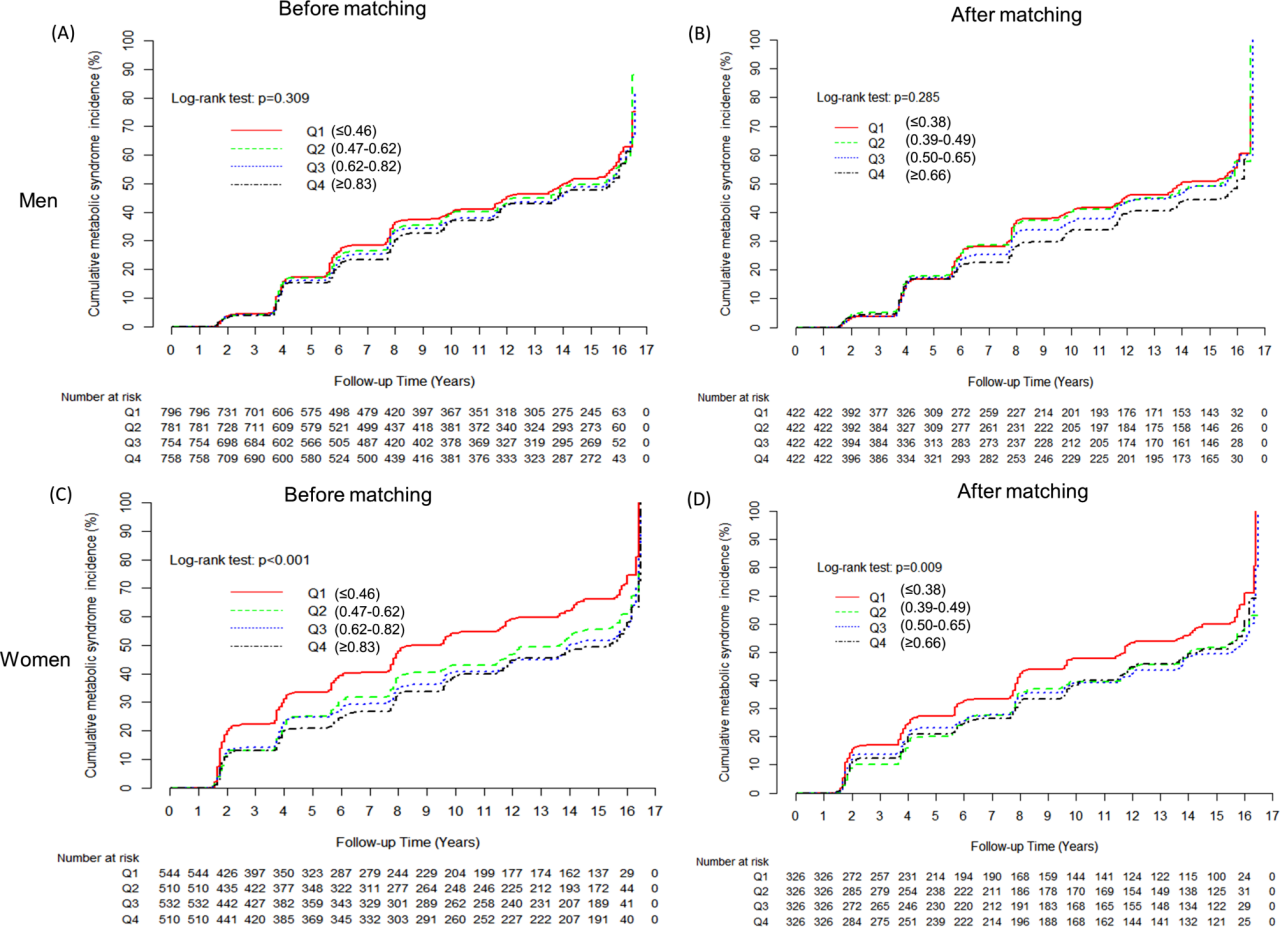
Cumulative incidence of MetS according to serum total bilirubin quartiles in men and in women before and after propensity score matching: (A) men before matching, (B) men after matching, (C) women before matching, and (D) women after matching

Table 3 presents the multivariate Cox proportional hazard regression analysis for the prediction of MetS by serum total bilirubin quartiles. With increasing serum total bilirubin quartile, the incidence rate per 1000 person-years proportionally decreased in both men and women. Before propensity score matching, the HRs (95% CIs) for incident MetS of the highest quartile in reference to the lowest quartile were 0.88 (0.75–1.04) for men and 0.75 (0.64–0.89) for women after adjusting for confounding factors. This association remained significant after the propensity score matching (adjusted HRs [95% CI] of the highest quartile were 1.00 [0.80–1.24] for men and 0.80 [0.65–0.99] for women). The cumulative incidences of MetS according to serum total bilirubin quartiles before and after propensity score matching are represented as Kaplan-Meier curves in Fig. 2. After matching, higher quartiles of serum total bilirubin showed significantly lower cumulative incidence of MetS in women (log-rank test *p* = 0.009), but not in men (log-rank test *p* = 0.285).

**Table 3:** Hazard ratios and 95% confidence intervals for incident metabolic syndrome by serum total bilirubin quartiles in men and women before and after propensity score matching

	Total bilirubin quartiles in men				Total bilirubin quartiles in women			
	Q1 (≤ 0.46)	Q2 (0.47–0.62)	Q3 (0.63–0.82)	Q4 (≥ 0.83)	Q1 (≤ 0.38)	Q2 (0.39–0.49)	Q3 (0.50–0.65)	Q4 (≥ 0.66)
**Before matching**								
Total, n	796	781	754	758	544	510	532	510
New cases of metabolic syndrome, n	371	360	336	327	342	275	262	248
Mean follow-up (years)	9.5 (5.3)	10.0 (5.2)	10.0 (5.2)	10.1 (5.2)	8.2 (5.6)	9.6 (5.6)	9.7 (5.7)	10.0 (5.5)
Person-years of follow-up	7572	7783	7559	7677	4480	4891	5139	5090
Incidence rate per 1000 person-year	49.0	46.3	44.5	42.6	76.3	56.2	51.0	48.7
Model 1	1.00 (ref)	0.96 (0.83–1.12)	0.94 (0.81–1.10)	0.93 (0.79–1.08)	1.00 (ref)	0.80 (0.68–0.94)	0.78 (0.66–0.93)	0.75 (0.64–0.89)
Model 2	1.00 (ref)	0.96 (0.83–1.11)	0.94 (0.80–1.09)	0.92 (0.78–1.08)	1.00 (ref)	0.79 (0.67–0.93)	0.78 (0.65–0.92)	0.75 (0.63–0.88)
Model 3	1.00 (ref)	0.95 (0.82–1.11)	0.88 (0.75–1.03)	0.88 (0.75–1.04)	1.00 (ref)	0.80 (0.67–0.94)	0.78 (0.68–0.95)	0.75 (0.64–0.89)
**After matching**								
Total, n	422	422	422	422	326	326	326	326
New cases of metabolic syndrome, n	197	195	189	174	194	165	157	165
Mean follow-up (years)	9.7 (5.3)	9.9 (5.3)	10.0 (5.2)	10.4 (5.2)	9.1 (5.6)	10.1 (5.5)	10.0 (5.6)	10.0 (5.5)
Person-years of follow-up	4103	4164	4203	4398	2969	3297	3248	3275
Incidence rate per 1000 person-year	48.0	46.8	45.0	39.6	65.3	50.0	48.3	50.4
Model 1	1.00 (ref)	1.00 (0.82–1.22)	0.98 (0.80–1.21)	0.88 (0.71–1.09)	1.00 (ref)	0.77 (0.62–0.96)	0.75 (0.60–0.93)	0.80 (0.65–0.99)
Model 2	1.00 (ref)	1.00 (0.82–1.22)	0.98 (0.80–1.20)	0.88 (0.71–1.09)	1.00 (ref)	0.77 (0.62–0.96)	0.74 (0.60–0.92)	0.80 (0.65–0.99)
Model 3	1.00 (ref)	1.03 (0.84–1.27)	1.01 (0.82–1.24)	1.00 (0.80–1.24)	1.00 (ref)	0.77 (0.62–0.96)	0.74 (0.59–0.92)	0.80 (0.65–0.99)

Model 1: adjusted for smoking status, regular drinker, and regular exercise

Model 2: adjusted for smoking status, regular drinker, regular exercise, and family history of diabetes

Model 3: adjusted for smoking status, regular drinker, regular exercise, family history of diabetes, HOMA-IR, and body mass index

## Discussion

Our study investigated the effect of bilirubin on risk of incident MetS in men and women after 16 years of longitudinal follow-up. Before propensity score matching, we observed that all components of MetS and insulin resistance in women were significantly inversely associated with total bilirubin. To minimize baseline disparities, we conducted propensity score matching for the components of MetS. Subsequently, we found that the higher quartiles of serum total bilirubin showed significantly lower cumulative incidence of MetS in women, but not in men.

Previous studies have shown conflicting results. The meta-analysis by Liang et al. in 2022 showed that there were conflicting results depending on the type of study conducted and from which sex the data were obtained. Most cross-sectional studies on the inverse association between serum total bilirubin and MetS presented results that were significant, but results of most cohort studies have not been as consistent as the cross-sectional studies [9, 11]. According to the meta-analysis by Liang et al. [9], the association between bilirubin level and MetS was significant in the combined pooled odds ratio (OR) of seven cross-sectional studies (OR = 0.91, 95% CI = 0.70–0.94, *P* = 0.005). However, the combined pooled OR of five cohort studies was not significant when confined to only men (OR = 0.91, 95% CI = 0.54–1.53, *P* = 0.72). In women only, the combined pooled OR of seven cross-sectional studies was significant (OR = 0.69, 95% CI = 0.57–0.84, *P* = 0.0002), but the combined pooled OR of two cohort studies was not significant (OR = 1.28, 95% CI = 0.60–1.02, *P* = 0.68). In five studies that were not stratified according to sex, the combined pooled OR was significant for an inverse association between bilirubin and MetS (OR = 0.75, 95% CI = 0.61–0.91, *P* = 0.004). Most cohort studies were conducted with small population numbers or short follow-up years that could have contributed to inconsistent results. Due to these contradictory results, our study was necessary in order to establish high quality evidence for the association between bilirubin level and MetS. To the best of our knowledge, our study is the first to prospectively follow-up a large number of participants longitudinally for 16 years in analyzing the association between bilirubin and MetS. Furthermore, our study is the only prospective cohort study that separately analyzed sex differences after conducting propensity score matching using a longitudinal data.

Although the precise mechanism behind the inverse association between bilirubin and MetS is not clear, some plausible explanations support our results. Bilirubin is the end product of heme catabolism, in which heme (iron-protoporphyrin IX) is an essential prosthetic group that includes hemoglobin, myoglobin, catalase, peroxidase, and mitochondrial cytochromes [18]. Heme oxygenase plays a key part in reducing reactive oxygen species (ROS) production through degradation of heme into bilirubin [19]. Numerous studies have already verified the antioxidant properties of bilirubin. Some studies demonstrated negative correlation of bilirubin with serum markers of oxidative stress [20, 21], as well as inflammatory markers such as C-reactive protein levels [22]. In addition, elevated bilirubin levels are being considered to have protective beneficial effects in diseases related to oxidative stress and chronic low-grade inflammation, such as CVD and T2DM [23–26], which include the metabolic abnormalities that begin with MetS. Furthermore, a recent discovery of a hormonal function of bilirubin has shown the ability of bilirubin to bind directly to peroxisome proliferator-activated receptor alpha (PPARα) that induces gene responses which eventually lead to improvement in insulin resistance and obesity [27]. For bilirubin to bind directly to its target PPARα, serum bilirubin concentration of EC_50_ = 9.0µM (0.53 mg/dl) is required [27, 28]. Our findings correlate with these published data because in both men and women the highest quartiles of bilirubin level that showed lowest risk of MetS were both over the established EC_50_ values (≥ 0.83 mg/dl or 14.2 µM for men, and ≥ 0.66 mg/dl or 11.3 µM for women).

To explain the discrepancy between men and women in the association between serum total bilirubin and MetS, sex hormone effects may play a role [29]. Park et al. found that serum bilirubin level was independently and inversely associated with testosterone deficiency, and deficiency of testosterone was also related to increased risk of MetS [30]. Another study demonstrated that estradiol and estrogen-receptors signaling facilitates bilirubin metabolism; as a result, women tended to experience shorter liver function recovery time than men after liver transplantation surgery [31]. Furthermore, a study by Balaz et al. showed that there are sex differences in the hepatic expression of heme oxygenase, in which women had a two-fold increase in hepatic heme oxygenase expression compared to men [32]. Due to sex differences in heme oxygenase activity, bilirubin may have different effects on men and women.

Some issues from our study remain unresolved. First, although our study included a substantial number of participants, our population consisted solely of individuals of Korean descent. Consequently, our findings might not be applicable to other ethnic groups. Second, the finding of sex differences in the association between bilirubin and MetS in our study was not validated with other cohorts. Third, serum bilirubin measurement was based on a single assessment because the bilirubin levels were not measured during the follow-up years, which may present a misclassification bias. Further studies with multiple cohorts and bilirubin levels during follow-up years are some additional factors that may validate our findings.

## Conclusion

In conclusion, serum total bilirubin levels were significantly inversely associated with MetS in women after propensity score matching, but there was no significant association observed in men. Sex differences in serum total bilirubin level should be noted when predicting the incidence of MetS in different sexes in clinical settings.

## Data Availability

The data used in our study are available from the corresponding authors upon reasonable request.

## Declarations

### Author details

Department of Family Medicine, Gangnam Severance Hospital, Yonsei University College of Medicine, Seoul, Korea.

## Abbreviations

ALT: alanine aminotransferase
AST: aspartate aminotransferase
BMI: body to mass index
BP: blood pressure
CI: confidence interval
CVD: cardiovascular disease
HbA1c: glycosylated haemoglobin
HDL: C-high-density lipoprotein cholesterol
HOMA: IR-homeostasis model assessment-insulin resistance
HR: hazard ratio
KoGES: Korea Centers for Disease Control and Prevention
LDL: C-low-density lipoprotein cholesterol
MetS: metabolic syndrome
OR: odds ratio
ROS: reactive oxygen species
T2DM: type 2 diabetes mellitus
WC: waist circumference
